# Intranasal inoculation of IFN-λ resolves SARS-CoV-2 lung infection via the rapid reduction of viral burden and improvement of tissue damage

**DOI:** 10.3389/fimmu.2022.1009424

**Published:** 2022-11-29

**Authors:** Haeun Shin, Sujin Kim, Ara Jo, Jina Won, Chan Hee Gil, So Yeon Yoon, Hyunkyung Cha, Hyun Jik Kim

**Affiliations:** ^1^ Department of Otorhinolaryngology, Seoul National University Hospital, Seoul, South Korea; ^2^ Seoul National University Hospital, Seoul, South Korea; ^3^ Sensory Organ Research Institute, Seoul National University Medical Research Center, Seoul, South Korea

**Keywords:** SARS-CoV-2, interferon-lambda, intranasal inoculation, viral clearance, lung remodeling

## Abstract

**Introduction:**

The innate immune responses of upper airway could further our understanding toward antiviral strategies against SARS-CoV-2. We characterize the potential of interferon (IFN)-λ as an innate immune inducer for the rapid clearance of SARS-CoV-2 in the lung and the therapeutic efficacy of intranasal inoculation of IFN-λ to resolve acute lung infection.

**Methods:**

Syrian golden hamsters were infected with SARS-CoV-2 and the dynamics of SARS-CoV-2 infection depending on IFN-λ inoculation were tested.

**Results:**

SARS-CoV-2-infected Syrian golden hamsters exhibited a significant decrease in body weight and high viral mRNA level at 3 days post-infection (dpi). Although viral replication was reduced completely from 7 dpi, the pathologic findings remained prominent until 14 dpi in the lung of hamsters. The transcription of IFN-λ was significantly induced in response to SARS-CoV-2 infection with the increase of IFN-stimulated genes. Intranasal inoculation of IFN-λ restricted SARS-CoV-2 replication in the lungs of infected completely from 3 dpi with markedly reduction of inflammatory cytokines. The transcriptional phenotypes were altered to the direction of damage repair and tissue remodeling in the lungs of SARS-CoV-2-infected hamsters following intranasal inoculation of IFN-λ, which improved SARS-CoV-2-caused lung damage.

**Conclusion:**

Collectively, our findings suggest that IFN-λ might be a potent innate immune inducer in the lung and intranasal inoculation of IFN-λ resolves SARS-CoV-2 infection with rapid viral clearance and improvement of lung damage.

## Introduction

Severe acute respiratory syndrome-related coronavirus-2 (SARS-CoV-2) leads to acute respiratory distress syndrome or viral pneumonia with severe damage to the lungs since 2019, and over 300 million people have suffered from a pandemic infection due to the coronavirus disease 2019 (COVID-19) (1, 2). To date, vaccine development to suppress COVID-19 has been progressing well, and the potential benefits of vaccination have been shown in reducing the spread of SARS-CoV-2. However, interest in effective SARS-CoV-2 therapeutics is increasing rapidly, and the emergence of SARS-CoV-2 variants has highlighted a need for new viral therapeutics (3, 4). Although the clinical use of corticosteroids and monoclonal antibodies became an important approach to COVID-19 therapy relatively early in the pandemic, those drugs can only be used limitedly depending on the host immune response, and a detailed understanding of the mechanisms behind the action of these treatments is still not fully developed. To devise successful therapeutic strategies against COVID-19, research on host-directed immune inducers against SARS-CoV-2 might be necessary; both the knowledge of the exact target organ for the delivery of immune inducers and the decision of the effective therapeutic substances to act on target organs are essential.

It is becoming increasingly apparent that nasal epithelial cells are the primary target of SARS-CoV-2, and the nasal epithelium is regarded as a portal for initial invasion or transmission of SARS-CoV-2 to the respiratory tract (5–7). In this regard, it is of immediate interest to determine whether localized suppression of SARS-CoV-2 in the nasal mucosa restricts viral spread to the respiratory tract and reduces the lung pathologies related to acute respiratory distress syndrome or viral pneumonia. Inhaled pathogens including respiratory viruses encounter the host immune system through the nasal passage, and the characteristics of the immune responses directly impact the initiation of the defense mechanism against the respiratory virus (7, 8). Thus, insights into the delivery of immune inducers to the nasal mucosa can provide fundamental information regarding the defense mechanisms against SARS-CoV-2 infection, and the induction of immune factors in the upper airway contributes to progress the development of new host-targeted therapeutics for respiratory virus control. These host-target immune inducers acting on the nasal mucosa can restrict SARS-CoV-2 infection more efficiently and prevent the progression to a pandemic infection when other variants emerge than drugs that directly target the virus.

The innate immune system of the respiratory epithelium serves as the first line of defense against respiratory viruses by producing interferon (IFN), a group of key molecules in the antiviral response (9–11). Traditionally, the antiviral innate immune response has been thought to be exclusively mediated by IFN-α and -βs followed by the adaptive immune response (12, 13). However, emerging evidence indicates that IFN-λs such as IFN-λ_1_, -λ_2_, -λ_3_, and -λ_4_ are likely to be mainly required for immune responses in the respiratory tract. In particular, IFN-λ has been shown to be the dominant IFN produced in the respiratory tract against respiratory viral infection and provides frontline protection against respiratory viruses that suppresses the initial viral spread in the respiratory epithelium (14, 15). Moreover, the IFN-λ-mediated innate immune response is necessary to protect the lungs from Influenza A virus infection beyond the antiviral properties of IFN-α and -βs (9, 15). Based on our previous data, IFN-λ is believed to be primarily responsible for the induction of local innate immune responses against viral invaders and to play an important role in restricting viral spread from the upper airway (10, 11). A recent study suggests that inhaled IFN-λ may have promise as a treatment for evolving SARS-CoV-2 variants (16) and contains similar results such as the route of administration of IFN-λ and the rapid suppression of viral replication *via* IFN-λ. In addition, although the results regarding upregulated Gene Ontology (GO) categories differ in some parts, RNA sequencing (RNA-seq) results also show a common feature compared with the present study in the increase in IFN-stimulated genes (ISGs) and induced genes related to the response to the virus. Our understanding about the improvement of SARS-CoV-2-induced lung infections *via* intranasal delivery of IFN-λ speculates on the challenges of therapeutic agents in COVID-19 as an immune inducer acting on the upper airway.

Here, we showed that intranasal inoculation of IFN-λ induced rapid SARS-CoV-2 clearance in the lungs of *in vivo* models and improved SARS-CoV-2-induced acute lung damages in concert with the transcriptional changes related to damage repair or tissue remodeling in the lung. The current findings provide evidence that IFN-λ might be a good candidate as an antiviral agent against SARS-CoV-2 and a potent inducer of host innate immune responses acting on the upper airway, and the signature of host responses from intranasal inoculation of IFN-λ could further our understanding of inhaled antiviral therapeutics against SARS-CoV-2.

## Materials and methods

### Viruses and reagents

A SARS-CoV-2 strain (BetaCov/Korea/SNU01/2020) was used in this study to induce an acute viral lung infection. Virus stocks were grown in Vero cells using a viral growth medium according to a standard procedure (17). Briefly, monolayers of Vero cells (ATCC^®^ CCL-81™) were inoculated with a viral stock, and then the cells were cultured at 37°C in a 5% carbon dioxide atmosphere. After 48 h of incubation, the supernatants were harvested by centrifugation at 5,000 rpm for 30 min to remove cellular debris. Virus stocks were titrated on Vero cells using a tissue culture infectious dose (TCID) assay (final concentration of virus stock: 5 × 10^6^ TCID50/ml) and stored at −80°C. Recombinant mouse IFN-λ_2_ (Accession #: NP_00101984) and IFN-λ_3_ (Accession #: NP_796370) were purchased from R and D Systems Inc. (Minneapolis, MN, USA).

### Hamsters and viral infection

The institutional review board (IRB) of the Seoul National University College of Medicine approved the protocol for this study (IRB #2020059). Experiments with Syrian golden hamsters were conducted at biosafety level-3 facilities in the Biomedical Research Institute of Seoul National University Hospital (bri.snuh.org) according to guidelines approved by the Institutional Animal Care and Use Committee (IACUC) of the Seoul National University College of Medicine (IACUC #20-0219-S1A0).

**Figure 1:**
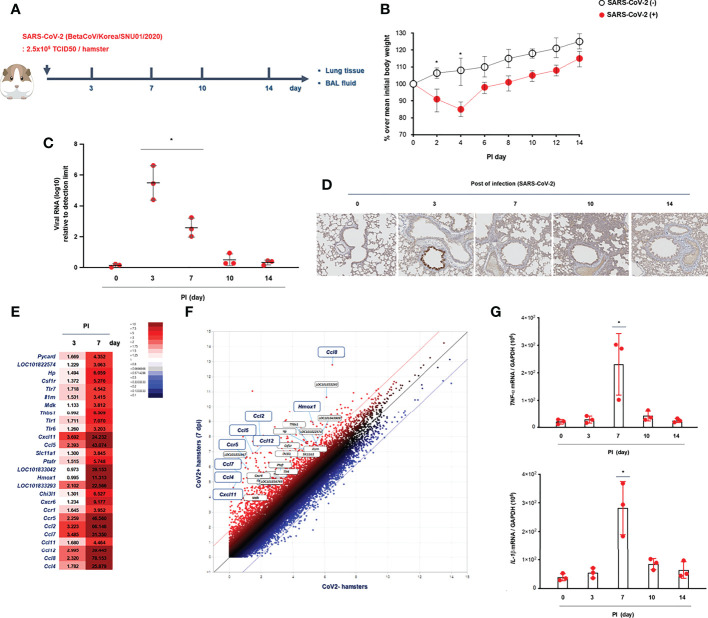
Kinetics of SARS-CoV-2 infection *in vivo*. **(A)** Schematic of the hamster model and experimental design for SARS-CoV-2 infection. Syrian golden hamsters were used in these experiments. The hamsters were infected with SARS-CoV-2 (BetaCoV/Korea/SNU01/2020, 2.5 × 10^5^ TCID50/hamster) at the indicated time points (N = 3 at each indicated time point). **(B)** The changes in the mean body weight of SARS-CoV-2-infected mice were compared with those of non-infected hamsters (analyzed by repeated-measures two-way ANOVA). **(C)** *Spike* mRNA levels in the lung tissue were determined at 0, 3, 7, 10, and 14 dpi. **(D)** Immunohistochemical analysis of spike protein using DAB chromogen was performed in lung sections from hamsters prior to and following SARS-CoV-2 infection (original magnification ×200). **(E, F)** Differentially expressed genes (DEGs) through bulk-seq using cell lysates from the lungs of SARS-CoV-2-infected hamsters were analyzed to further characterize the transcriptional alteration in response to SARS-CoV-2. The scatter plots indicating DEGs correlated with “inflammatory responses.” **(G)** mRNA levels of *IL-1β* and *TNF-α* were determined in the lungs of SARS-CoV-2-infected hamsters at 0, 3, 7, 10, and 14 dpi. Real-time PCR results are analyzed by Mann–Whitney U test and presented as mean ± SD values from three independent experiments. *p < 0.05 vs. non-infected hamsters.

Male Syrian golden hamsters (Orientalbio, Seoul, Korea) aged 7 weeks were used as a wild-type (WT) animal model. To induce viral infections, WT hamsters were inoculated with SARS-CoV-2 [2.5 × 10^5^ TCID50/hamster in 50 μl of phosphate-buffered saline (PBS)] by intranasal delivery to induce acute viral lung infections (**Figure 1A**). After euthanizing the hamsters, we obtained the lung tissue and it was used in real-time polymerase chain reaction (PCR), bulk RNA sequencing (bulk-seq), and immunohistochemistry analyses.

### Real-time PCR

Lung tissue was obtained from hamsters infected with SARS-CoV-2 for 3, 7, 10, and 14 days, and total RNA was isolated using TRIzol (Invitrogen). cDNA was synthesized from 3 μg of RNA with random hexamer primers and Moloney murine leukemia virus reverse transcriptase (PerkinElmer Life Sciences, Waltham, MA, USA, and Roche Applied Science, Indianapolis, IN, USA). Amplification was performed using the TaqMan Universal PCR Master Mix (PE Biosystems, Foster City, CA, USA) according to the manufacturer’s protocol. Briefly, amplification reactions had a total volume of 12 μl and contained 2 μl of cDNA (reverse transcription mixture), oligonucleotide primers (final concentration of 800 nM), and TaqMan hybridization probe (200 nM). Real-time PCR probes were labeled at the 5’ end with carboxyfluorescein and at the 3’ end with the quencher carboxytetramethylrhodamine.

To quantify the cellular viral level and host gene expression, cellular RNA was used to generate cDNA. The viral RNA level of SARS-CoV-2 was monitored using quantitative PCR for the s*pike* gene with forward and reverse primers and probe of 5’-ATTCAAGACTCACTTTCTTCCACAG-3’, 5’-TGTTTAAAGCTTGTGCATTTTGGTTGA-3’, and 5’-CACATCTTGAAGTTTTCC-3’, respectively. Viral RNA of SARS-CoV-2 was quantified using primers to the *spike* gene, and viral RNA per ng total RNA is graphed as fold change from the limit of detection (40 cycles of PCR) as 2^40-Ct^. Primers for hamster *TNF-α*, *IL-1β*, *IFN-β*, *IFN-λ_2/3_*, *IFN-γ*, *Mx1*, *IFIT3*, *Rsad2*, *Spp1*, *Saa3*, and *Fabp4* were purchased from Applied Biosystems (Foster City, CA, USA). Real-time PCR was performed using a PE Biosystems ABI PRISM^®^ 7700 Sequence Detection System. The thermocycling parameters were as follows: 50°C for 2 min, 95°C for 10 min, and then 40 cycles of 95°C for 15 s and 60°C for 1 min. Target mRNA levels were quantified using target-specific primer and probe sets for *Rdrp*, *TNF-α*, *IL-1β*, *IFN-β*, *IFN-λ_2/3_*, *IFN-γ*, *Mx1*, *Mx1*, *IFIT3*, *Rsad2*, *Spp1*, *Saa3*, and *Fabp4*. All real-time PCR data were normalized to the level of glyceraldehyde phosphate dehydrogenase (1 × 10^6^ copies) to correct for variations between samples.

### Immunohistochemistry and histologic analysis

Lung tissue was fixed in 10% (vol/vol) neutral buffered formalin and embedded in paraffin. Paraffin-embedded tissue slices were stained with hematoxylin and eosin (H&E) solution (Sigma-Aldrich, St. Louis, MO, USA). Histopathologic analysis of inflammatory cells in H&E-stained lung sections was performed in a blinded fashion using a semiquantitative scoring system described previously (18). Lung sections from at least three hamsters were examined, and pathologic findings were scored from 0 to 3 points depending on the inflammatory cell infiltration of lung tissue as follows: 0, normal; 1, a ring of inflammatory cells one layer deep; 2, a ring of inflammatory cells two to four cells deep; and 3, a ring of inflammatory cells more than four cells deep. The histological score for the PBS/PBS control hamster lung tissue was always 0. At least five separate areas were assessed from similar sections within each hamster, and at least three hamsters in each condition were assessed. Inflammatory cell infiltration was counted by an examiner who was blinded to the experimental group and is expressed as the number of cells per high-power field.

### Subjects and clinical review

We recruited 223 patients confirmed to have COVID-19 at Seoul National University Hospital (Seoul, Korea) in 2021 and reviewed their medical records, including the diagnostic techniques used for COVID-19 and their clinical symptoms. SARS-CoV-2 infection was confirmed in patients who complained of fever, sore throat, and cough, and *Rdrp* mRNA was detected in a throat swab through real-time PCR in those patients. Patients hospitalized for COVID-19 underwent chest X-ray at least twice during their hospitalization, and they were discharged when a real-time PCR test was negative and their main symptoms had improved (**Table 1**).

**Table 1:** Clinical characteristics of COVID-19 patients (N = 223).

Characteristics		Values
Mean age in years		60.2
Range in years		39-85
Mean BMI (kg/m^2^)		28.5
Gender		Number of patients (%)
	Male	132 (59.2)
	Female	91 (40.8)
Clinical features		Number of patients (%)
General symptoms	Fever (°C)	
	37.6-38.0	28 (12.5)
	39.0-39.0	87 (39.1)
	> 39.0	49 (22.0)
	Headache	40 (17.9)
	Sore throat	53 (23.7)
	Myalgia	67 (30.0)
Respiratory symptoms	Cough	165 (73.9)
	Sputum	121 (54.2)
	Dyspnea	132 (59.2)
	Chest pain	21 (9.4)
Nasal symptoms	Nasal obstruction	8 (3.6)
	Hyposmia	22 (9.8)
	Runny nose	20 (8.9)
Chest X-ray		Number of patients (%)
Normal		24 (10.8)
Abnormal	Improved	59 (26.5)
	Not improved	140 (62.7)

BMI, body mass index.

### Bulk RNA sequencing

#### RNA isolation

Total RNA was isolated using TRIzol reagent (Invitrogen). RNA quality was assessed by Agilent 2100 bioanalyzer using the RNA 6000 Nano Chip (Agilent Technologies, Amstelveen, Netherlands), and RNA quantification was performed using ND-2000 Spectrophotometer (Thermo Inc., DE, USA).

#### Library preparation and sequencing

For control and test RNAs, the construction of the library was performed using QuantSeq 3’ mRNA-Seq Library Prep Kit (Lexogen, Inc., Austria) according to the manufacturer’s instructions. In brief, each total RNA was prepared, an oligo-dT primer containing an Illumina-compatible sequence at its 5’ end was hybridized to the RNA, and reverse transcription was performed. After degradation of the RNA template, second-strand synthesis was initiated by a random primer containing an Illumina compatible linker sequence at its 5’ end. The double-stranded library was purified by using magnetic beads to remove all reaction components. The library was amplified to add the complete adapter sequences required for cluster generation. The finished library was purified from PCR components. High-throughput sequencing was performed as single-end 75 sequencing using NextSeq 500 (Illumina, Inc., USA).

### Data analysis

QuantSeq 3’ mRNA-seq reads were aligned using Bowtie2 (19). Bowtie2 indices were generated from either the genome assembly sequence or the representative transcript sequences for aligning to the genome and transcriptome. The alignment file was used for assembling transcripts, estimating their abundances and detecting differential expression of genes. Differentially expressed genes (DEGs) were determined based on counts from unique and multiple alignments using coverage in Bedtools (20). The Read Count (RC) data were processed based on the TMM+CPM normalization method using EdgeR within R (R Development Core Team, 2020) using Bioconductor (21). Gene classification was based on searches done using Database for Annotation, Visualization and Integrated Discovery (DAVID) (http://david.abcc.ncifcrf.gov/) and Medline databases (http://www.ncbi.nlm.nih.gov/). Data mining and graphic visualization were performed using ExDEGA (Ebiogen Inc., Korea). DEGs were defined as those adjusted *p* < 0.05, fold change ≥2.0 or <2.0, and normalized data (log2) ≥2.0 or <2.0, and the original bulk-seq data for hamster models with *SARS-CoV-2 infection* are available at https://www.ncbi.nlm.nih.gov/geo/query/acc.cgi?acc=GSE204696.

### Statistical analyses

A representative experiment result (three hamsters for each group) was described as a graph among three repeated experiments. We present the *in vivo* results of real-time PCR as mean ± SD values from three individual hamsters, and differences between treatment groups were evaluated by repeated-measures two-way analysis of variance (ANOVA), and two-sample t-testing was used to analyze the bulk-seq data. GraphPad Prism (version 8; GraphPad Software, La Jolla, CA, USA) was used for all of the statistical analyses, and differences were considered significant at *p*-value <0.05.

## Results

### The kinetics of SARS-CoV-2 infection in an *in vivo* lung

Initially, Syrian golden hamsters (N = 15) were infected with SARS-CoV-2 (CoV2+ hamster) *via* intranasal administration (**Figure 1A**). As gross determinants of virus-induced morbidity, the body weights and survival rate of CoV2+ hamsters were monitored at 0, 3, 7, 10, and 14 days post-infection (dpi). The mean body weight of CoV2- hamsters was 112.5 g, and a significant drop in the body weight of CoV2+ hamsters was shown from 2 dpi until 4 dpi. Then, the body weights of the CoV2+ hamsters recovered gradually with a 100% survival rate until 14 dpi, but the difference in weight was maintained for up to 14 dpi compared to CoV2- hamsters (**Figure 1B**). The real-time PCR results reveal that the mean level of *Rdrp* RNA was significantly elevated in the lungs of the CoV2+ hamsters, with the highest viral RNA level observed at 3 dpi and then decreased gradually from 7 dpi (**Figure 1C**). An immunohistochemistry analysis of the SARS-CoV-2 spike protein using 3,3’-diaminobenzidine (DAB) chromogen revealed that the number of DAB stain–positive cells was significantly increased at 3 dpi and dominantly observed in bronchial epithelial cells (**Figure 1D** and **Supplementary Figure S1**). As a next step, we investigated DEGs in bulk-seq data from lung lysates of CoV2+ hamsters to further characterize transcriptional alterations in response to SARS-CoV-2. Compared with CoV2- hamsters, we found 32,315 DEGs based on the cutoff criteria in the lungs of CoV2+ hamsters at 3 and 7 dpi. The associated GO term “inflammatory responses” was examined first, and 60 relevant DEGs were observed in the lung of CoV2+ hamsters, especially at 7 dpi (**Figure 1E**). Scatter plot data for genes associated with “inflammatory responses” revealed that *Cxcl11*, *Ccl5*, *Hmox1*, *Ccr5*, *Ccl2*, *Ccl4*, *Ccl7*, *Ccl8*, and *Ccl12* mRNA levels were higher in the lungs of CoV2+ hamsters (**Figure 1F**). Although the bulk-seq data did not show a significant increase, the real-time PCR results revealed that mRNA levels of *TNF-alpha* and *IL-1beta* (**Figure 1G**) also increased in the lungs of CoV2+ hamsters, and the highest levels were observed at 7 dpi. These data suggest that SARS-CoV-2 infection reaches its most significant acute phase in hamsters within 7 days after infection with the induction of inflammatory cytokines.

### The clinical symptoms and chest pathologies of the hospitalized COVID-19 patients

**Figure 2:**
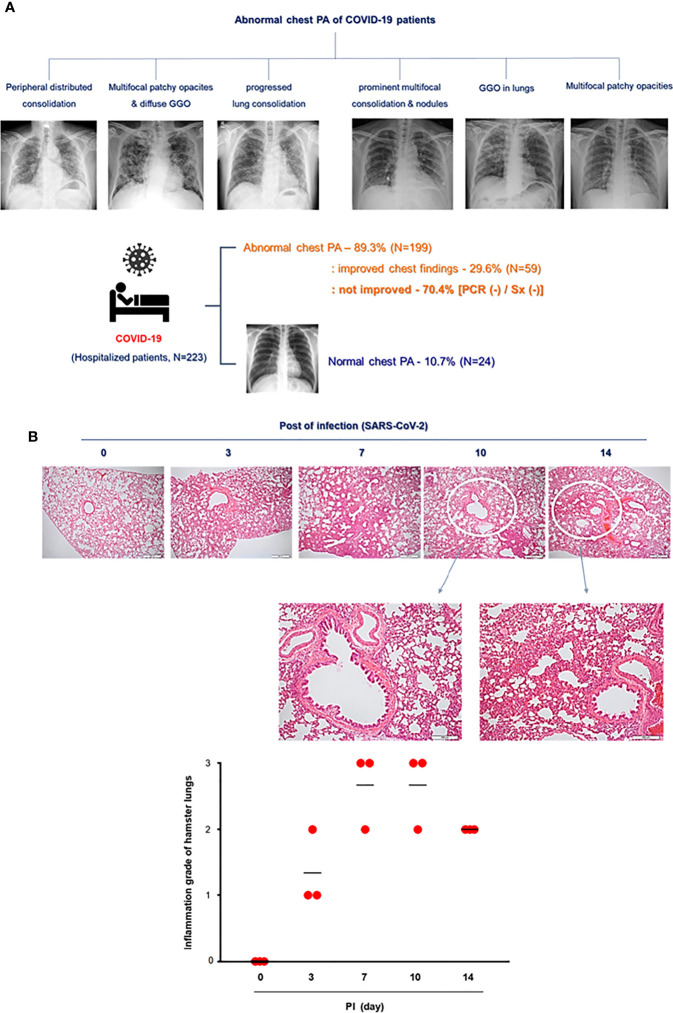
Lung histopathologic findings in COVID-19 patients and CoV2+ hamsters. **(A)** The clinical analysis of hospitalized COVID-19 patients (N = 223) and the findings of chest PA in COVID-19 patients with abnormal chest x-ray readings until discharge. Syrian golden hamsters were used in these experiments. The hamsters were infected with SARS-CoV-2 (BetaCoV/Korea/SNU01/2020, 2.5 × 10^5^ TCID50/hamster) at the indicated time points (N = 3 at each indicated time point). **(B)** H&E-stained micrographs were also generated from lung sections of hamsters obtained at indicated time points of SARS-CoV-2 infection (scale bar = 100 µM). Micrographs shown are representative of lung sections from three hamsters and were used to assess the inflammation and tissue damage and to calculate a histological score.

To compare the pathologic findings shown in the animal model with those in COVID-19-confirmed patients, the clinical data of COVID-19 patients were reviewed focusing on chest pathologic findings and the period of clinical symptoms. The hospitalized COVID-19 patients (N = 223) who tested *Rdrp* RNA-positive were divided into three groups: first, patients with normal chest PA findings at admission (normal); second, patients with abnormal chest PA at admission but completely improved by discharge (improved); and third, patients had an abnormal chest PA at admission and did not improve upon discharge (not improved). The average length of hospitalization of the patients was 11.3 days, and at the time of admission, the average *Rdrp* CT value was 21.17. They received combined treatment with antibiotics, oral steroids, and remdesivir. If symptoms improved completely and PCR results were negative, the patient was discharged. Clinical data showed that fever, cough, and sputum were representative COVID-19-related symptoms and lasted longer than other symptoms in COVID-19 patients (**Table 1** and **Supplementary Figure S2**). Among 223 patients, 24 had normal chest PA at the time of admission, but in 199 patients, chest PA readings were abnormal findings such as ground-glass opacity, consolidation, multifocal patch opacities, and prominent consolidation and nodules (**Figure 2A**). Interestingly, 29.6% of patients with pathologic chest findings had marked improvement in chest PA upon discharge, but the chest PA findings were still abnormal in 70.4% of patients when they were discharged, although fever, sore throat, and cough were completely resolved within 12 days and their PCR results turned negative (**Table 1**). Although viral replication was below the detectable level in the lungs of CoV2+ hamsters after 10 dpi, the difference in weight was maintained for up to 14 dpi (**Figure 1B**). Extensive inflammatory cell infiltration around the bronchioles was observed in H&E-stained micrographs of lung sections in CoV2+ hamsters, and mean values of the histologic scores had not reduced by 14 dpi (**Figure 2B**). These findings revealed that the abnormal lung inflammation persisted in COVID-19 patients even if their symptoms were completely improved and PCR results were negative after treatment, and this distinctive characteristic was also evident in the lungs of CoV2+ hamsters. Therefore, therapeutic selection for the improvement of lung lesions and rapid viral clearance are likely to be clinically important in the treatment of COVID-19 patients.

### Rapid induction of IFN-λ in the lung of hamsters in response to SARS-CoV-2 infection

**Figure 3:**
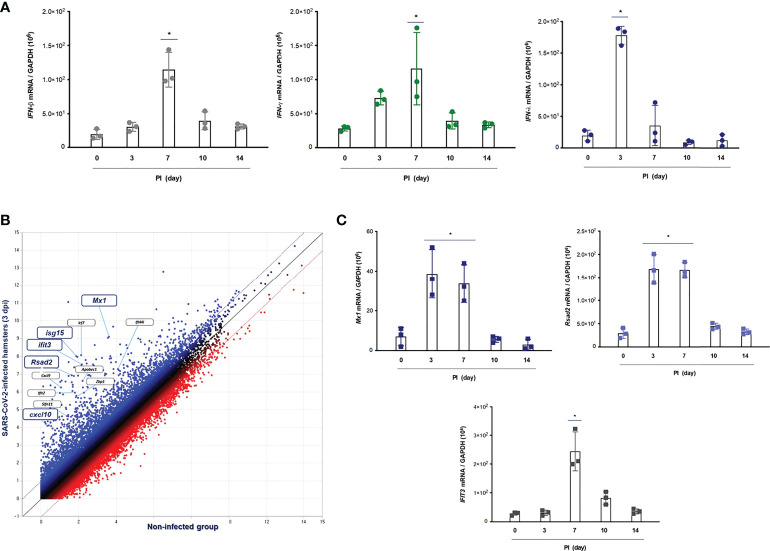
IFN-related immune responses in the lung of CoV2+ hamsters. Syrian golden hamsters were used in these experiments. The hamsters were infected with SARS-CoV-2 (BetaCoV/Korea/SNU01/2020, 2.5 × 10^5^ TCID50/hamster) at the indicated time points (N = 3 at the indicated time points). **(A)** The transcriptions of *IFN-β*, *IFN-γ*, and *IFN-λ_2/3_* were determined in the lungs of SARS-CoV-2-infected hamsters at 0, 3, 7, 10, and 14 dpi. **(B)** The scatter plot data showed DEGs associated with “response to IFNs” in the lungs of SARS-CoV-2-infected hamsters at 3 dpi. **(C)** *Mx1*, *Rsad2*, and *Ifit3* mRNA levels were determined in the lungs of hamsters in response to SARS-CoV-2 infection at 0, 3, 7, 10, and 14 dpi. Real-time PCR results are analyzed by Mann–Whitney U test and presented as mean ± SD values from three independent experiments. *p < 0.05 vs. non-infected hamsters.

To assess the distinctive pattern of IFN expression and secretion in the lung of hamsters after SARS-CoV-2 infection, Syrian golden hamsters (N = 3 per each group) were infected with SARS-CoV-2 *via* intranasal administration, and then, we measured the mRNA levels of *IFN-α*, *IFN-β_1_*, *IFN-λ_2/3_*, and *IFN-γ* at 0, 3, 7, 10, and 14 dpi by real-time PCR. The result revealed that the mRNA level of *IFN-λ_2/3_* was significantly higher at 3 dpi and reduced gradually onward until 14 dpi. Both *IFN-β*_1_ and *IFN-γ* transcriptions were induced in the lung of CoV2+ hamsters, and the highest elevation was observed at 7 dpi. By contrast, the mRNA level of *IFN-α* was not induced after SARS-CoV-2 infection in the lung of hamsters (**Figure 3A**). Next, we investigated the DEGs, the GO term associated “response to virus,” and 49 DEGs were observed in the lung of CoV2+ hamsters, especially 3 and 7 dpi. The scatter plot data revealed that transcriptions of *Mx1*, *Rsad2* (*Viperin*), *Irf7*, *Cxcl10*, *Isg15*, and *Ifit3* were significantly higher in the lung of CoV2+ hamsters from 3 dpi (**Figure 3B**). The real-time PCR data showed that *Mx1*, *Rsad2*, and *Ifit3* mRNA levels were significantly elevated in the lung of hamsters in response to SARS-CoV-2 infection from 3 dpi (**Figure 3C**). We speculated that these ISGs including *Mx1*, *Rsad2*, and *IFIT3* were markedly induced at 3 dpi in the lung of CoV2+ hamsters and their transcriptions might be regulated by IFN-λ signaling based on the time point of ISG induction. Collectively, we found that transcriptions of IFN-λ and ISGs were induced rapidly in *in vivo* lung after SARS-CoV-2 infection, and they might be involved in the antiviral innate immune in the lung at the early stage of SARS-CoV-2 infection.

### Intranasal inoculation of IFN-λs restricts SARS-CoV-2 infection in the lung of hamsters

**Figure 4:**
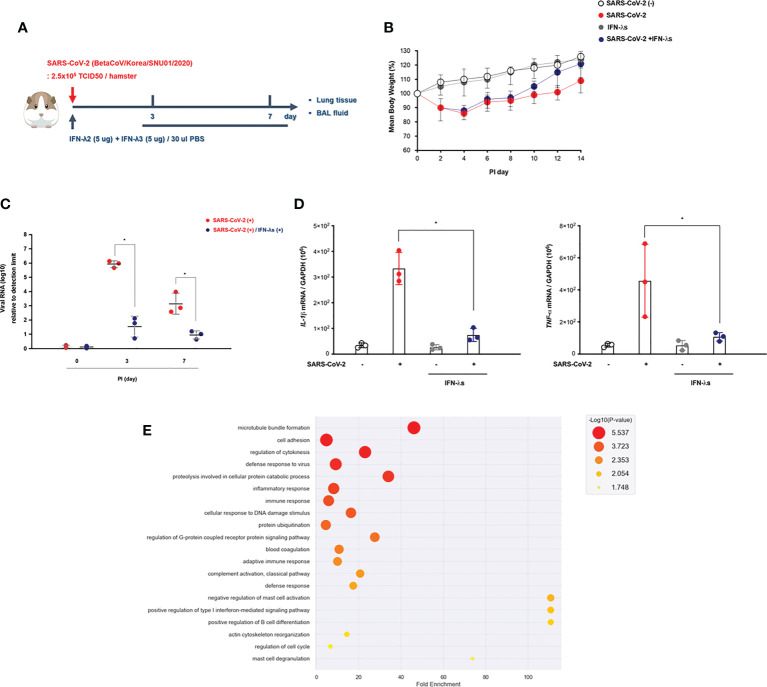
Intranasal inoculation of IFN-λ promotes the viral clearance of SARS-CoV-2. **(A)** Schematic of the hamster model and experimental design for SARS-CoV-2 infection and intranasal inoculation of IFN-λ. Syrian golden hamsters were infected with SARS-CoV-2 [3 dpi (N = 3), 7 dpi (N = 3)] and concomitantly treated with recombinant IFN-λ (IFN-λ_2_: 5 μg, IFN-λ_3_: 5 μg with PBS 30 μl) at 3 and 7 dpi [CoV2- (N = 3)/CoV2+ 3 dpi (n = 3)/CoV2+ 3 dpi with IFN-λ (N = 3)/CoV2+ 7 dpi (N = 3)/CoV2+ 7 dpi with IFN-λ (N = 3)]. **(B)** Changes in the mean body weights of the hamsters were compared according to SARS-CoV-2 infection and intranasal inoculation of IFN-λ. **(C)** *Spike* RNA levels in the lung tissue were determined at 0, 3, and 7 dpi. **(D)** The mRNA levels of both *IL-1β* and *TNF-α* in response to IFN-λ were determined in the lungs of SARS-CoV-2-infected hamsters at 0, 3, and 7 dpi. **(E)** Dot plot visualization of enriched GO terms in the lung lysates of SARS-CoV-2-infected hamsters that received an intranasal inoculation of IFN-λ. The real-time PCR results were analyzed by the Mann–Whitney U test and are presented as mean ± SD values from three independent experiments. *p < 0.05 vs. non-infected hamsters.

We next sought to explore the antiviral properties of intranasal inoculation of IFN-λ in the lung of CoV2+ hamsters. The nasal cavities of Syrian golden hamsters (N = 3 in each of the four groups) were inoculated with recombinant IFN-λ (IFN-λ_2_: 5 µg, IFN-λ_3_: 5 µg with 30 ul PBS) immediately after SARS-CoV-2 infection (**Figure 4A**). Then, the alteration of *Rdrp* RNA level and improvement of histopathologic findings in response to IFN-λ were determined at 0, 3, and 7 dpi in the lung of hamsters.

We found that the weight recovery of CoV2+ hamsters treated with IFN-λ was more remarkable after 7 dpi (**Figure 4B**), and the real-time PCR results revealed that *spike* RNA levels were significantly decreased in the lung of CoV2+ hamsters at 3 dpi with inoculation of IFN-λ (**Figure 4C**). Both *IL-1β* and *TNF-α* gene expressions were also significantly reduced in the lung of CoV2+ hamsters in response to intranasal inoculation of IFN-λ (**Figure 4D**).

As compared with CoV2- hamsters, there were 3,498 DEGs based on the cutoff criteria in the lung of SARS-CoV-2-infected hamsters depending on intranasal inoculation of IFN-λ at 3 and 7 dpi. We performed GO enrichment analysis by DAVID using lung lysates from CoV2+ hamsters with intranasal inoculation of IFN-λ to confirm the effect of IFN-λ in restricting SARS-CoV-2 infection. The GO analysis showed that DEGs linked with biological processes were mainly enriched in “adaptive immune response,” “cell adhesion,” “proteolysis involved in cellular protein catabolic process,” “innate immune response,” “defense to virus,” and “T cell costimulation” (**Figure 4E**). These findings suggest that intranasal inoculation of IFN-λ demonstrates a potential clearance of SARS-CoV-2 and inflammation in the lungs of hamsters at an early stage of infection with induction of immune responses.

### Intranasal inoculation of IFN-λs leads to a strong induction of IFN-stimulated genes in the lungs of hamsters

**Figure 5:**
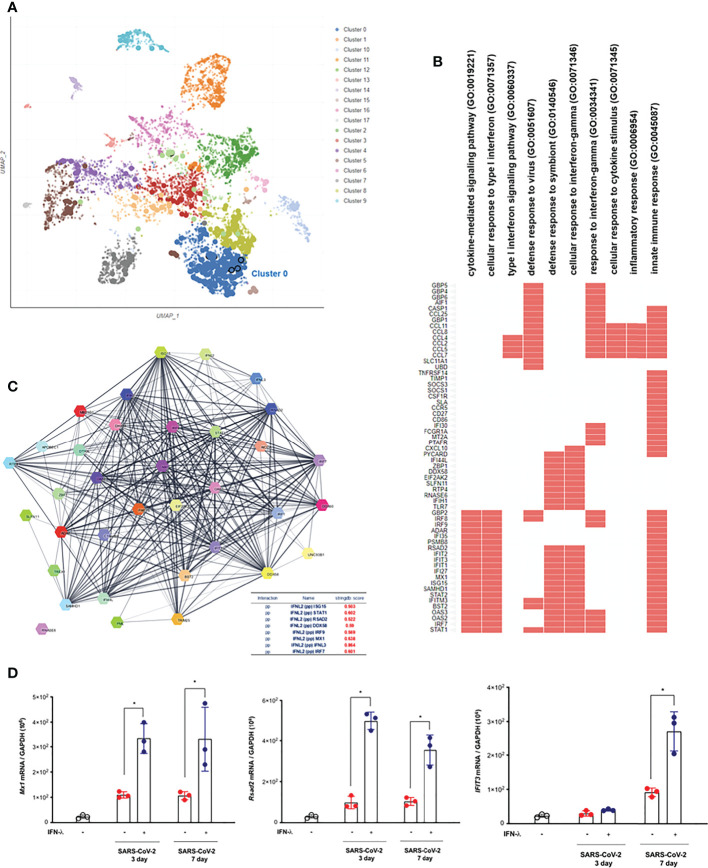
Intranasal inoculation of IFN-λs leads to a strong induction of IFN-stimulated genes (ISGs) in the lungs of CoV2+ hamsters. Syrian golden hamsters were infected with SARS-CoV-2 and concomitantly treated with recombinant IFN-λ (IFN-λ_2_: 5 μg, IFN-λ_3_: 5 μg with PBS 30 μl) at 3 and 7 dpi [CoV2- (N = 3)/CoV2+ 3 dpi (n = 3)/CoV2+ 3 dpi with IFN-λ (N = 3)/COV2+ 7 dpi (N = 3)/CoV2+ 7 dpi with IFN-λ (N = 3)]. EnrichR analysis was performed to determine the DEGs in the lungs of CoV2+ hamsters depending on the inoculation of IFN-λs. **(A)** The scatter plot of EnrichR data revealed that 3,498 DEGs were classified into 18 clusters linked to biological process, and the top 10 significant GO terms were included into cluster 0. **(B)** The GO terms in cluster 0 were plotted according to their *p*-value ranking. **(C)** Protein–protein interaction (PPI) network of the DEGs linked to “response to IFN” indicates the PPI among IFN-λ and the ISGs (PP: protein*–*protein). **(D)** *Mx1*, *Rsad2*, and *Ifit3* mRNA levels were determined by real-time PCR at 3 and 7 dpi in SARS-CoV-2-infected lungs from animals that also received an intranasal inoculation of IFN-λ. The real-time PCR results were analyzed by the Mann–Whitney U test and are presented as mean ± SD values from three independent experiments. *p < 0.05 vs. non-infected hamsters.

In order to understand the transcriptional dynamics and influence on IFN-λ inoculation in greater detail, we also performed EnrichR analysis using bulk-seq data. The scatter plot revealed that 3,498 DEGs were classified into 18 clusters, and the top 10 significant GO categories linked with biological processes were included into cluster 0 (**Figure 5A**). GO results showed that DEGs of cluster 0 were mainly enriched in “cytokine-mediated signaling pathway,” “cellular response to type I interferon,” “type I interferon signaling pathway,” and “defense response to virus” (**Figure 5B** and **Table 2**). We also found that the transcriptions of *Mx1*, *Rsad2*, *Isg15*, *IFIT1*, *IFIT2*, *IFIT3*, *Stat1*, *Stat2*, and *BST2* were rapidly induced in the lung of CoV2+ hamsters in response to IFN-λ inoculation at 3 dpi (**Figure 5B**). A protein–protein interaction (PPI) network of 33 DEGs linked with the response to IFN was constructed using the Search Tool for the Retrieval of Interacting Genes/Proteins (STRING) online database and visualized by Cytoscape. Connectivity map analysis revealed the more characteristic PPI between Ifnl2 and Ifnl3 (STRING database score: 0.864), and Mx1, Stat1, IRF7, Ddx58, Rsad2, and Isg15 showed a more significant influence on maintaining the stability of the PPI network with IFN-λ_2_ (**Figure 5C**). In addition, real-time PCR results showed that the transcription of ISGs such as *Mx1*, *Rsad2*, and *IFIT3* significantly increased at 3 and 7 dpi, respectively, in the lung of CoV2+ hamsters after intranasal inoculation of IFN-λ compared to only CoV2+ hamsters (**Figure 5D**). We found that intranasal inoculation of IFN-λ would be effective in inducing the transcription of ISGs at an early stage of infection in concert with the restriction of SARS-CoV-2 replication in the lung.

**Table 2:** Top 10 significant gene ontology (GO) biological process including differentially expressed gene (DEGs) in the lung of SARS-CoV_2-infected hamsters after inoculation of IFN-λs at 3 and 7 dpi.

Term	Overlap genes
cytokine-mediated signaling pathway (GO:0019221)	*IFITM3, CD86, CXCL9, F13A1, SLA, ADAR, IFI35, IFIT1, IFI30, CXCL2, IFIT3, CXCL5, IL18BP, IFIT2, LMNB1, PYCARD, MT2A, CASP1, TIMP1, CCR5, RSAD2, IFI27, OAS2, OAS3, PSME1, IRF7, BIRC5, IRF8, IRF9, CSF1R, CCL11, PSMD13, PTAFR, CSF2RB, IL2RG, SAMHD1, LRP8, PSMB10, SOCS3, CCL8, SOCS1, CCL7, CCL5, CCL4, HMOX1, CCL2, TNFRSF14, GBP2, FCGR1A, GBP1, CCL25, STAT1, STAT2, MX1, ISG15, PSMB8, PSMB9, VEGFA, BST2, CXCL10, CXCL11, SAA1, CD27*
cellular response to type I interferon (GO:0071357)	*IFITM3, RSAD2, STAT1, STAT2, MX1, ADAR, ISG15, IFI35, IFIT1, SAMHD1, IFIT3, PSMB8, IFIT2, BST2, IFI27, OAS2, OAS3, IRF7, IRF8, GBP2, IRF9*
type I interferon signaling pathway (GO:0060337)	*IFITM3, RSAD2, STAT1, STAT2, MX1, ADAR, ISG15, IFI35, IFIT1, SAMHD1, IFIT3, PSMB8, IFIT2, BST2, IFI27, OAS2, OAS3, IRF7, IRF8, GBP2, IRF9*
defense response to virus (GO:0051607)	*IFITM3, RTP4, IFIT1, SAMHD1, IFI44L, IFIT3, IFIT2, PYCARD, IFIH1, ZBP1, RSAD2, DDX58, STAT1, STAT2, MX1, RNASE6, EIF2AK2, ISG15, BST2, CXCL10, SLFN11, IFI27, OAS2, OAS3, IRF7, TLR7*
defense response to symbiont (GO:0140546)	*IFITM3, RTP4, IFIT1, SAMHD1, IFI44L, IFIT3, IFIT2, PYCARD, IFIH1, ZBP1, RSAD2, DDX58, STAT1, STAT2, MX1, RNASE6, EIF2AK2, ISG15, BST2, SLFN11, IFI27, OAS2, OAS3, IRF7, TLR7*
cellular response to interferon-gamma (GO:0071346)	*GBP6, CCL25, GBP5, CCL11, STAT1, PTAFR, IFI30, AIF1, MT2A, CCL8, CCL7, OAS2, OAS3, CCL5, CCL4, CASP1, IRF7, IRF8, CCL2, FCGR1A, GBP2, GBP1, IRF9, GBP4*
response to interferon-gamma (GO:0034341)	*IFITM3, GBP6, CCL25, GBP5, CCL11, STAT1, SLC11A1, AIF1, BST2, CCL8, CCL7, CCL5, UBD, CCL4, CASP1, IRF8, CCL2, GBP2, GBP1, GBP4*
cellular response to cytokine stimulus (GO:0071345)	*CD86, CSF1R, CCL11, PTAFR, F13A1, SLA, CSF2RB, IL2RG, IFIT1, LRP8, AIF1, CXCL2, IL18BP, PYCARD, SOCS3, MT2A, CCL8, SOCS1, CCL7, CCL5, CCL4, CASP1, HMOX1, CCL2, TIMP1, GBP2, CCR5, GBP1, GBP4, GBP6, CCL25, GBP5, STAT1, STAT2, PYHIN1, MAPK13, VEGFA, CXCL10, SAA1, HCLS1, BIRC5, IRF8, CHI3L1, MNDA*
inflammatory response (GO:0006954)	*CSF1R, CXCL9, CCL11, HP, AIF1, CXCL2, THBS1, CXCL5, FUT7, CCL8, CCL7, CCL5, CCL4, C3AR1, NLRP3, CCL2, CCR5, CCL25, SLC11A1, TLR1, CXCL10, CXCL11, KRT16, CHI3L1, SIGLEC1, FCGR2B, NAIP*
innate immune response (GO:0045087)	*IFITM3, SHMT2, SLA, ADAR, TREM2, IFIT1, IFIH1, CLEC7A, UBD, DHX58, RPL39, DDX58, SLC11A1, MX1, RNASE6, ISG15, BST2, CXCL10, IFI27, SLPI, KRT16, CLEC6A, SAA1, IRF7, CD300E, CLEC4E, CFB*

### Intranasal inoculation of IFN-λ resolves SARS-CoV-2-induced lung damages at an early stage of infection

**Figure 6:**
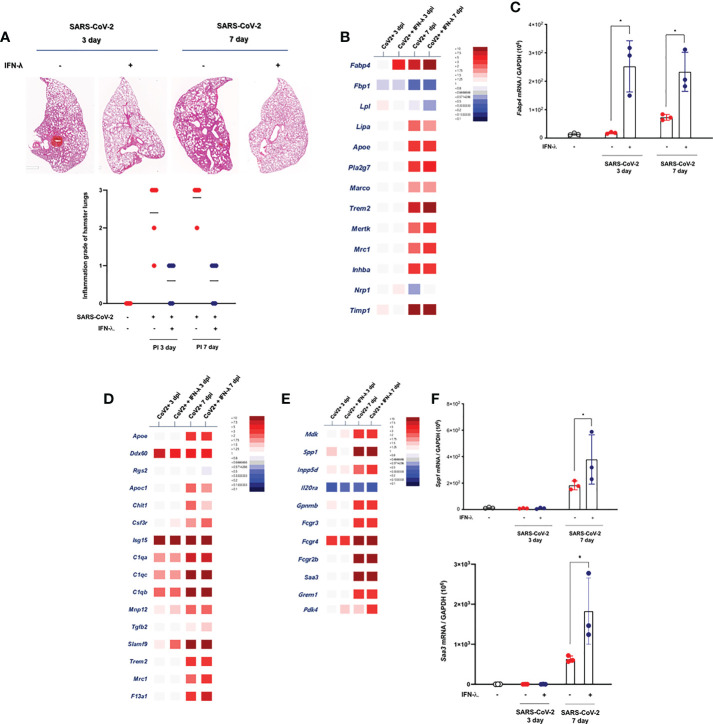
Intranasal inoculation of IFN-λ resolves the lung damages in CoV2+ hamsters. **(A)** Lung tissue samples were obtained from SARS-CoV-2-infected hamsters at 3 and 7 dpi, and the whole-lung microscopic findings were compared according to IFN-λ inoculation [CoV2- (N = 3)/CoV2+ 3 dpi (N = 3)/CoV2+ 3 dpi with IFN-λ (N = 3)/COV2+ 7 dpi (N = 3)/CoV2+ 7 dpi with IFN-λ (N = 3)]. **(B)** DEGs associated with tissue-resident alveolar MФ were examined. Thirteen DEGs related to anti-inflammation and lipid metabolism were determined using bulk-seq data. **(C)** *Fabp4* mRNA level was determined by real-time PCR at 3 and 7 dpi in the lungs of CoV2+ hamsters that also received an intranasal inoculation of IFN-λ. **(D)** DEGs associated with the activation of mononuclear phagocytes were examined, and 16 DEGs were observed in the lungs of CoV2+ hamsters, especially at 3 and 7 dpi. **(E)** DEGs associated with the regulation of tissue remodeling were examined, and 11 DEGs were observed in the lungs of CoV2+ hamsters, especially at 7 dpi. **(F)** Both *Spp1* and *Saa3* mRNA levels were determined by real-time PCR at 3 and 7 dpi in the lungs of CoV2+ hamsters that also received an intranasal inoculation of IFN-λ. The H&E micrographs shown are representative of lung sections from three hamsters and were used to assess inflammation and tissue damage and calculate a histological score. Real-time PCR results were analyzed by the Mann–Whitney U test and are presented as mean ± SD values from three independent experiments. *p < 0.05 vs. non-infected hamsters.

As described above, prolonged tissue damage in the lung might be a critical feature of SARS-CoV-2 infection, and efforts to resolve the extensive lung injury are aimed at pathogenetic treatment in COVID-19 patients. It has been suggested that the severe lung injury observed in some patients with COVID-19 might be a consequence of the hyperactivated immune system rather than of inadequate viral clearance (22), and we determined the change of histopathologic inflammatory features in the lung of CoV2+ hamsters depending on intranasal inoculation of IFN-λ. As compared with only CoV2+ hamsters, intranasal inoculation of IFN-λ also resulted in significantly attenuated pathologic findings in the lungs of SARS-CoV-2-infected hamsters from 3 dpi, with significantly lower histologic scores, and a clear improvement of pathologic findings was also observed in the lung of CoV2+ hamsters until 7 dpi (**Figure 6A**).

The mechanism of SARS-CoV-2-induced histopathologic lung damage remains incompletely understood, but recently, the pathological changes in SARS-CoV-2-infected lung might be hallmarks of the severity of COVID-19, and an enrichment of fibrosis-associated gene signatures was a suggestive mechanism for prolonged lung damage in COVID-19 subjects (22). To further determine the association of IFN-λ inoculation and improvement of histopathologic findings in the lung of SARS-CoV-2-infected hamsters, we assessed the alteration about transcriptional phenotypes of lung tissue through bulk-seq data and investigated the DEGs that are more dominantly expressed in the lung of SARS-CoV-2-infected hamsters in response to IFN-λ. The bulk-seq results showed that the transcription of *Fabp4* was highly induced in the lung of SARS-CoV-2-infected hamsters in response to intranasal inoculation of IFN-λ (**Figure 6B**). In particular, *Fabp4* gene expression was significantly induced (6.34-fold over control) at 3 dpi, and it was more elevated in the lung of CoV2+ hamsters that received intranasal IFN-λ inoculation at 7 dpi (13.31-fold over control). *Fabp4* is characterized to mediate lipid handling and metabolism and has been proven to upregulate CD8+ tissue-resident memory T cells of alveolar macrophage (MФ) after acute viral infection (18), and our real-time PCR data also showed that CoV2+ hamsters that received IFN-λ inoculation exhibited more induction of *Fabp4* at 3 and 7 dpi (**Figure 6C**). *Trem2* might be associated with apoptotic cell uptake, and *Timp1* is a protease inhibitor involved in tissue remodeling and fibrosis (23). Our bulk-seq data revealed that the transcriptions of both genes were induced in the lung of CoV2+ hamsters with intranasal inoculation of IFN-λ. The bulk-seq data showed that genes expressed in mononuclear phagocytes (22) such as *Ddx60*, *C1qa*, *C1qb*, *C1qc*, *Slamf9*, *Mrc1*, and *F13a1* were highly induced in the lungs of CoV2+ hamsters in response to intranasal inoculation of IFN-λ, and the induction of *F13a1* (7.6-fold at 7 dpi over control) and *Slamf9* (2.6-fold at 3 dpi, 46.1-fold at 7 dpi over control) was more significant in the lungs of CoV2+ hamsters in response to IFN-λ inoculation (**Figure 6D**). In addition, 11 DEGs related to “regulation of tissue remodeling” were observed in the lungs of CoV2+ hamsters, and the transcriptions of *Spp1* (137.4-fold over control), *Saa3* (92.2-fold over control), and *Fcrg4* (64.6-fold over control) were particularly induced by an intranasal inoculation of IFN-λ (**Figure 6E**). Similar to real-time PCR results, IFN-λ-regulated induction of these genes for tissue remodeling was prominent in the lung of CoV2+ hamsters at 7 dpi (**Figure 6F**).

Together, these results suggest that intranasal inoculation of IFN-λ improved the pathologic findings of SARS-CoV-2-infected lungs at an early stage of infection and induced the transcriptional alteration of genes related to damage repair, lipid metabolism, mononuclear phagocytes, and tissue remodeling in the lung of CoV2+ hamsters.

## Discussion

Our study reveals that IFN-λ is a potent innate immune inducer acting on the nasal mucosa and is responsible for the regulation of antiviral mechanisms in the SARS-CoV-2-infected lung *in vivo* at an early stage of SARS-CoV-2 infection. Intranasal inoculation of IFN-λ promotes rapid clearance of SARS-CoV-2 and triggers lipid metabolism, tissue repair, or remodeling-associated transcriptional profiles in SARS-CoV-2-infected lungs in concert with the induction of ISGs. This study highlights the possibility of IFN-λ as a host-directed innate immune inducer acting on the nasal mucosa to restrict SARS-CoV-2 replication with the improvement of tissue damage and hyperinflammation in the lung of SARS-CoV-2 infection.

We are currently facing the devastating outbreak of COVID-19, and the development of therapeutic agents for COVID-19 patients and vaccines to suppress the spread of SARS-CoV-2 is being made in various ways (2–4). According to several clinical data, COVID-19 patients exhibit a very high viral titer in the upper airway at the early stages of infection before symptoms appear, and they can spread the virus quickly to others in the asymptomatic state (24, 25). While existing neutralizing antibodies and vaccines against the spike protein of SARS-CoV-2 have conferred protection for many individuals, there is an urgent need for a therapeutic agent that acts rapidly in the upper airway of COVID-19 patients and suppresses the spread of SARS-CoV-2 quickly at an early stage of infection.

Human respiratory viruses first encounter host defense mechanisms in the nasal mucosa, and a specialized innate immune system can be activated at the nasal epithelium that capable of combating invasion by respiratory viruses (26–29). In particular, growing evidence shows that the entry factors for SARS-CoV-2, including angiotensin-converting enzyme 2 (ACE2) and transmembrane serine protease 2 (TMPRSS2), are dominantly found in the nasal epithelial cells, and the nasal epithelium has been determined as a potential cellular target of SARS-CoV-2 infection (7, 30–32). SARS-CoV-2 infection might be initiated at the nasal epithelium and progresses from the upper airway to the lung if the host immune mechanism does not protect against SARS-CoV-2 infection efficiently. A specialized innate immune system exists at the nasal epithelium to combat invasion by SARS-CoV-2, and protective innate immune responses lower the burden of disease in the infected host by increasing antiviral resistance and disease tolerance (7, 22, 33). We thought that it would be more effective to use an antiviral substance that activates the innate immune responses rapidly in the nasal mucosa to reduce the higher viral titer of SARS-CoV-2 in the upper airway of COVID-19 patients at an early stage of infection.

Innate immune responses are mediated by an increase in IFN secretion that contributes to the viral clearance in nasal or respiratory epithelium (7, 9, 11), and both IFN-α and -β are well documented to mediate the innate immune response and regulate the subsequent activation of the adaptive immune system against SARS-CoV-2 (6, 13). However, our previous studies demonstrated that IFN-λ might be more critical for the innate immune response against respiratory viral infection in the nasal epithelium, and inhaled delivery of IFN-λs was more effective in controlling acute viral lung infection in animal models (10, 11). Moreover, IFN-λ deficiency has been linked to higher susceptibility to the fatal respiratory virus infection, but compensation of IFN-λ can protect the susceptible host from respiratory virus spread (34). We hypothesized that this intimate association of IFN-λ could potentially benefit the host respiratory tract and restrict SARS-CoV-2 replication through the rapid upregulation of the innate immune response at the level of the nasal mucosa. In addition, the research on the therapeutic application of IFN-λ against acute SARS-CoV-2-induced lung infection will enable a greater understanding of host-directed defense strategies in the respiratory tract.

The present study showed that IFN-λ was more dominantly induced in the lung of SARS-CoV-2-infected hamsters from an early stage of infection and mediated the induction of ISGs such as *Mx1*, *Rsad2*, and *IFIT3* preferentially from 3 dpi. Interestingly, even though the IFN-β and IFN-γ transcriptions were induced in an *in vivo* lung in response to SARS-CoV-2 infection, both genes’ expressions were relatively higher from 7 dpi and viral clearance occurred from 3 dpi with significant weight recovery in hamsters. We propose that the elevated levels of IFN-λ at an early time point after SARS-CoV-2 infection constitute the primary antiviral defense in an *in vivo* lung, and IFN-λ plays a novel role in protecting the respiratory tract from SARS-CoV-2 infection.

The current data showed that intranasal inoculation of IFN-λs completely inhibited SARS-CoV-2 in the respiratory tract from acute-phase infection, as demonstrated by the significantly decreased viral mRNA levels in the lung of CoV2+ hamsters. Furthermore, intranasal inoculation of IFN-λs significantly induced innate immune responses such as the transcription of ISGs resulting in limiting the replication of SARS-CoV-2. This study establishes the importance of IFN-λ as an innate immune inducer against acute SARS-CoV-2 infection and demonstrates that intranasal inoculation of IFN-λ exerts rapid therapeutic effects against SARS-CoV-2 at the early stage of infection, even though it would be delivered into the nasal mucosa.

While the pathophysiology of SARS-CoV-2-induced lung damage and respiratory diseases remains incompletely understood, about 5% of COVID-19 patients develop acute respiratory lung disease including severe viral pneumonia and acute respiratory distress syndrome, which requires prolonged respiratory supports and is associated with serious mortality (21, 35, 36). It has been repeatedly reported that a subset of COVID-19 patients develops a detrimental hyperinflammatory condition in their lungs, and acute lung injury due to SARS-CoV-2 is driven by inappropriate immune responses (17). In the current findings, rapid viral clearance occurs after 3 dpi, and the body weight returns to the normal range 5 days after SARS-CoV-2 infection, but the abnormal histopathologic findings of *in vivo* lungs did not improve even 14 days after infection. Therefore, we aimed to prove the therapeutic possibility of IFN-λ to alter the phenotypes of cellular compartments and hyperinflammatory conditions in the lungs after SARS-CoV-2 infection.

Supporting this notion, it has been suggested that aberrant activation of immune cells and pro-inflammatory polarization of alveolar MФ might be related to dysregulated local and mucosal inflammatory responses in the lung of susceptible COVID-19 patients (34, 35). In addition, several clinical data proposed that circulating monocyte numbers might be introduced as prognostic biomarkers in lung damage of COVID-19 patients, and the hyperinflammation and tissue fibrosis in the lung have been associated with poor prognosis of the lung infection in COVID-19 (36, 37). Therefore, we understand that the switch of transcriptional phenotypes to damage repair or anti-inflammation combined with rapid viral clearance promotes the improvement of histopathologic findings and hyperinflammation in the SARS-CoV-2-infected lung. Although we did not elucidate a very specific linkage mechanism and cell-specific pattern in the present study, an interesting point that we noticed among the results of this study is that SARS-CoV-2-induced lung infection was completely improved and transcriptional phenotypes related to tissue repair were altered in an *in vivo* lung with intranasal inoculation of IFN-λ. Our RNA-seq data reveal that IFN-λ increased the transcriptions of genes that might be involved in lipid surfactant metabolism, damage repair, and anti-inflammatory responses in the lung. In particular, IFN-λ inoculation markedly elevated *Fabp4* and *Slamf9* transcriptions in the lungs of CoV2+ hamsters from an early stage of infection. We determined that an intranasal inoculation of IFN-λ strengthened the frontline innate immune responses rapidly in the upper airway and could boost immune responses to tissue repair, thereby improving the lung damage caused by SARS-CoV-2.

Recently, Mo-MФ and alveolar MФ may be considered an emerging cellular target for therapeutic interventions against virus-induced extensive tissue damage in the lung that might be more typical clinical findings in COVID-19 (37, 38), and we did not prove a definite interaction between Mo-MФ or alveolar MФ and IFN-λ in the lung of CoV2+ hamsters. However, we found that intranasal inoculation of IFN-λ induced the transcription of *Spp1* and Saa3 in the lung of CoV2+ hamsters, which might be involved in the regulation of tissue remodeling. Therefore, we estimated that the rapid antiviral effect of intranasal inoculation of IFN-λ might be useful in restricting the transmission of SARS-CoV-2 between hosts in the early stages of infection, and it will be a more potent therapeutic means to resolve the lung damages fundamentally after SARS-CoV-2 infection. In addition to the current results of transcriptional alterations induced by IFN-λ, we need to proceed with single-cell RNA-seq demonstrating the interaction between innate immune cells including MФ and neutrophils and IFN-λ in the lung after respiratory virus infection.

In summary, our data show that the IFN-λ-mediated innate immune response is dominantly induced in an *in vivo* lung after SARS-CoV-2 infection, and an intranasal inoculation of IFN-λ resulted in a rapid viral clearance and complete improvement of extensive lung infections caused by SARS-CoV-2. Our results deepen the understanding about the strength of rapid innate immune inducers as inhaled antivirals and suggest that IFN-λ will be an even more potent therapeutic means against SARS-CoV-2 infections to overcome the limitations of corticosteroids and neutralizing antibodies.

## Data availability statement

The datasets presented in this study can be found in online repositories. The name of the repository and accession number can be found below: National Center for Biotechnology Information (NCBI) Gene Expression Omnibus (GEO), https://www.ncbi.nlm.nih.gov/geo/, GSE167509.

## Ethics statement

The institutional review board (IRB) of the Seoul National University College of Medicine approved the protocol for this study (IRB #2020059). The patients/participants provided their written informed consent to participate in this study. Experiments with Syrian golden hamsters were conducted at biosafety level-3 facilities in the Biomedical Research Institute of Seoul National University Hospital (bri.snuh.org) according to guidelines approved by the Institutional Animal Care and Use Committee (IACUC) of the Seoul National University College of Medicine (IACUC #20-0219-S1A0).

## Author contributions

HS and HJK conceived the study and designed the experiments. HS, SK, SYY, and HC carried out the study, including sample collection and sample preparation. JW, CHG, and AJ performed additional work, design, and data analysis. HS and HJK drafted the manuscript. All authors contributed to the article and approved the submitted version.

## Funding

This work was supported by the Basic Science Research Program through the National Research Foundation of Korea, funded by the Ministry of Education (2019M3C9A6091945 and 2022R1A2C2011867 awarded to HJK. This research was also supported by a grant from the Korean Health Technology R&D Project through the Korean Health Industry Development Institute, funded by the Ministry of Health & Welfare of the Republic of Korea (HI20C0546) awarded to HJK.

## Conflict of interest

The authors declare that the research was conducted in the absence of any commercial or financial relationships that could be construed as potential conflicts of interest.

## Publisher’s note

All claims expressed in this article are solely those of the authors and do not necessarily represent those of their affiliated organizations, or those of the publisher, the editors and the reviewers. Any product that may be evaluated in this article, or claim that may be made by its manufacturer, is not guaranteed or endorsed by the publisher.
