# Supplementary material for: Intranasal inoculation of IFN-λ resolves SARS-CoV-2 lung infection via the rapid reduction of viral burden and improvement of tissue damage
Source: Front Immunol. 2022 Nov 29;13:1009424. doi: 10.3389/fimmu.2022.1009424 (PMC9744928; doi:10.3389/fimmu.2022.1009424)

Supplementary Material

# Supplementary Figures and Tables

**Supplementary Figure S1.** Immunohistochemical analysis of the spike protein using DAB chromogen was performed in lung sections from hamsters (N=3) at 3 days after SARS-CoV-2 infection (CoV2+) (scale bar 100 μm).


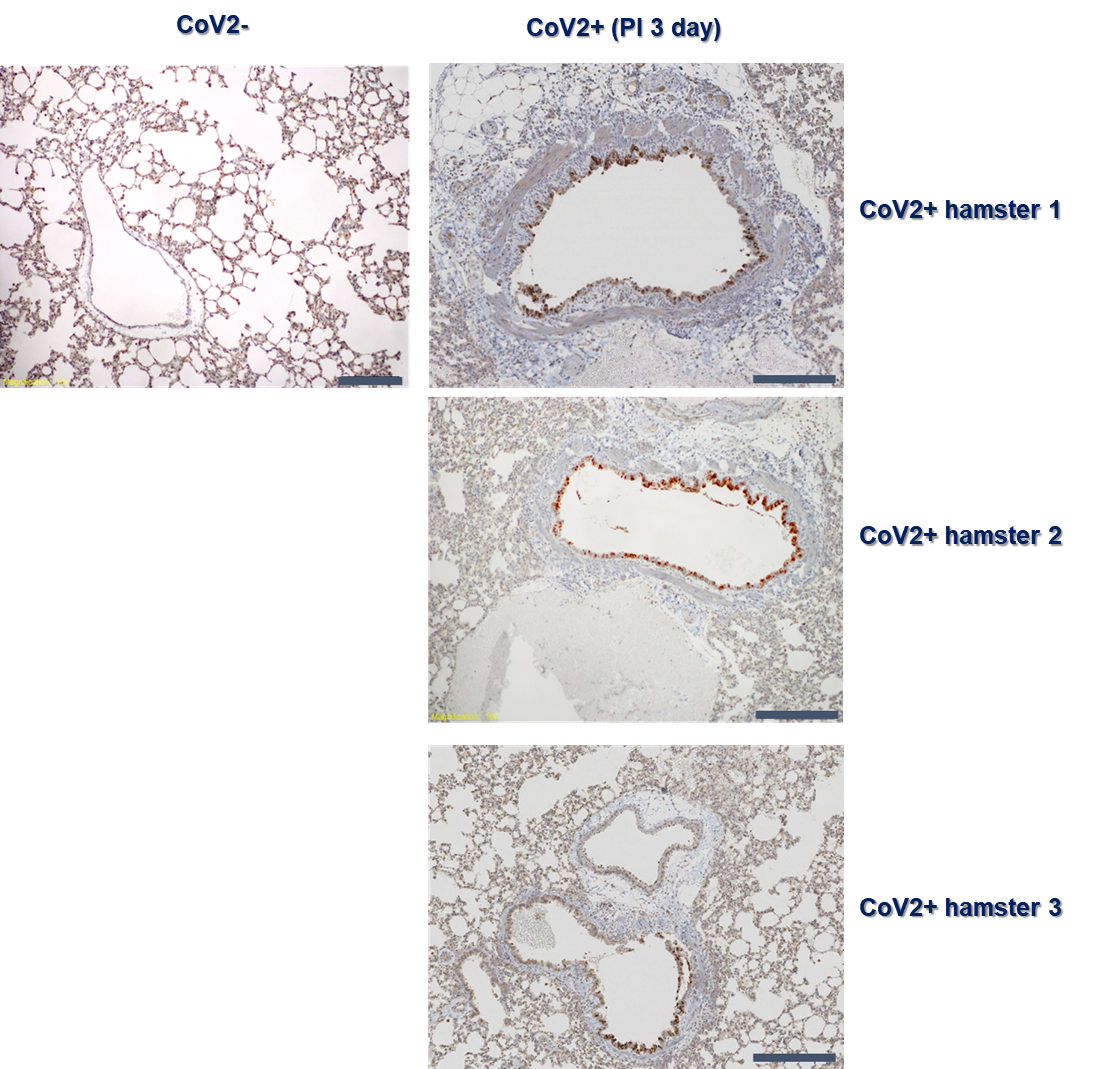


**Supplementary Figure S2.** Schematic illustration depicts the summarized review of medical records in patients who have been diagnosed with COVID-19 and have been hospitalized for respiratory symptoms or fever.


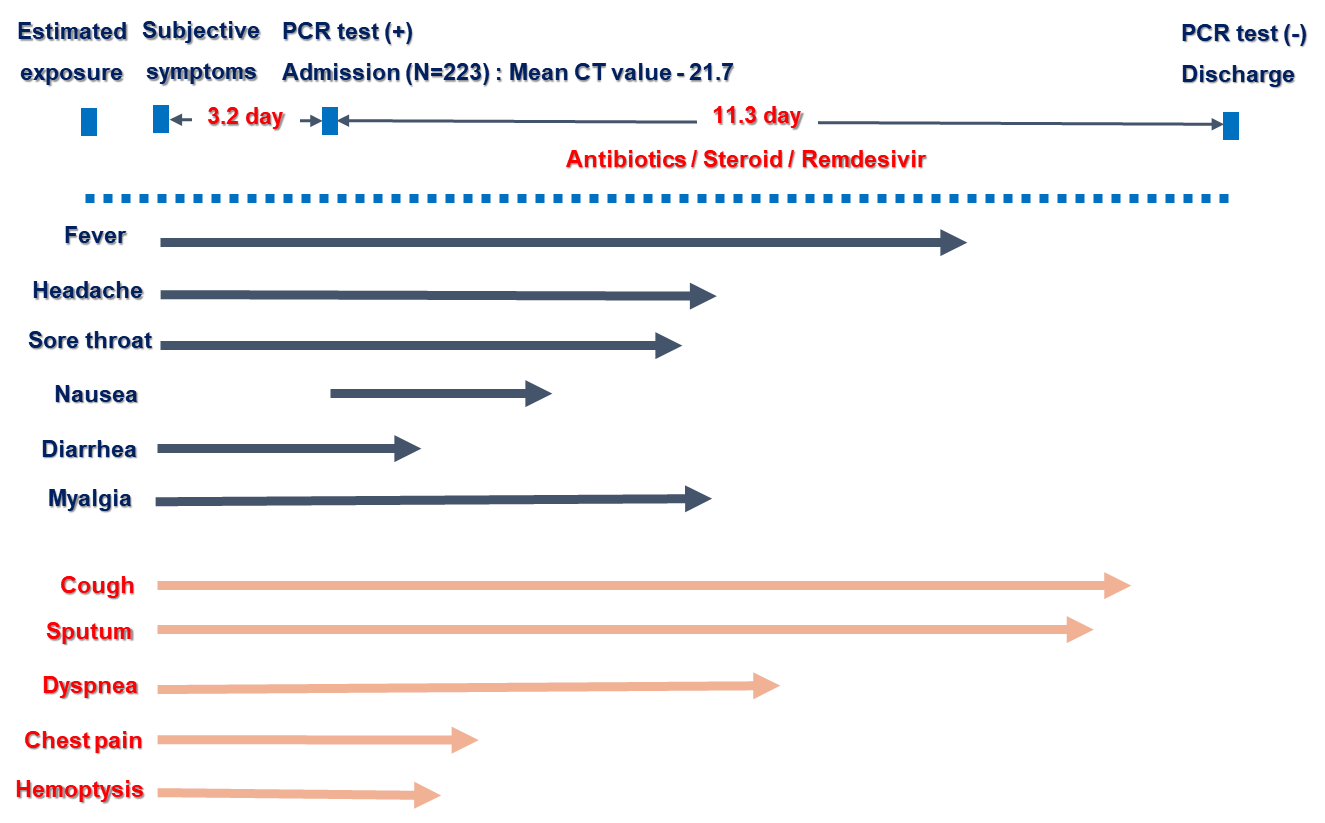

Supplement: Supplementary file 1 [file DataSheet_1.doc]
